# Multiple Damaged Cables Identification in Cable-Stayed Bridges Using Basis Vector Matrix Method

**DOI:** 10.3390/s23020860

**Published:** 2023-01-11

**Authors:** Jianying Ren, Xinqun Zhu, Shaohua Li

**Affiliations:** 1 State Key Laboratory of Mechanical Behavior and System Safety of Traffic Engineering Structures, Shijiazhuang Tiedao University, Shijiazhuang 050043, China; 2 Department of Engineering Mechanics, Shijiazhuang Tiedao University, Shijiazhuang 050043, China; 3 School of Civil and Environmental Engineering, University of Technology Sydney, Ultimo, NSW 2007, Australia

**Keywords:** multiple damaged cables identification, basis vector matrix, bending strain, finite element model, cable-stayed bridge

## Abstract

A new damaged cable identification method using the basis vector matrix (BVM) is proposed to identify multiple damaged cables in cable-stayed bridges. The relationships between the cable tension stiffness and the girder bending strain of the cable-stayed bridge are established using a force method. The difference between the maximum bending strains of the bridges with intact and damaged cables is used to obtain the damage index vectors (DIXVs). Then, BVM is obtained by the normalized DIXV. Finally, the damage indicator vector (DIV) is obtained by DIXV and BVM to identify the damaged cables. The damage indicator is substituted into the damage severity function to identify the corresponding damage severity. A field cable-stayed bridge is used to verify the proposed method. The three-dimensional finite element model is established using ANSYS, and the model is validated using the field measurements. The validated model is used to simulate the strain response of the bridge with different damage scenarios subject to a moving vehicle load, including one, two, three, and four damaged cables with damage severity of 10%, 20%, and 30%, respectively. The noise effect is also discussed. The results show that the BVM method has good anti-noise capability and robustness.

## 1. Introduction

Cable-stayed bridges are widely used around the world due to their rigid stiffness, good aerodynamics, high seismic resistance, and desired aesthetics [1]. Like other types of bridge structures, cable-stayed bridges deteriorate over time due to damage accumulation caused by their aging and operational loading. If the damage cannot be identified early, the deterioration or damage accumulation of the bridges will lead to the collapse of entire structures and result in devastating human fatalities and substantial economic losses. Structural health monitoring is critical to prevent catastrophic structure collapse and provide quantitative information for effective and economic structural maintenance. In the bridge health monitoring system, one of the most important techniques is to identify structural damage. The structural damage detection methods could be regarded as the core of SHM strategies [2].

In the last several decades, many studies have been conducted on structural damage identification for cable-stayed bridges. In general, there are mainly two types of damage in the cable-stayed bridge to be identified. One is damage of the girders. The other is damage of stayed cables. For the girder damage of the cable-stayed bridge, vibration modal parameters are widely used to identify structural damage, and many methods have been developed, such as the enhanced coordinate modal assurance criterion (ECOMAC), damage index method (DI), mode shape curvature method (MSC), and modal flexibility index method (MFI) [3]. A probabilistic neural network with modal frequency data was used to predict damage locations in the Ting Kau Bridge by Zhou et al. [4]. When the noise level is less than 10%, the damage type and region can be identified with high confidence (greater than 85%) using the first 20 modes. The method cannot identify the multi-damage situations. Based on the three-dimensional multi-scale FE models of the Runyang Bridge, the performance of some damage location methods is evaluated by Ding et al. [5], including a modal curvature index, a modal strain energy index, and a modal flexibility index. The relative modal flexibility change (RFC) between intact and damage states was formulated as an index to locate damage in the Ting Kau Bridge by Ni et al. [6]. Without the environmental effects, the RFC index performs well for locating damage in single-damage cases. For multi-damage cases, the RFC index may provide false damage identification at members with low sensitivity. A technique to reduce the limitations of modal identification in damage detection using reduced field data for nondestructive structural health monitoring of a cable-stayed concrete bridge was designed by Ismail et al. [7]. The method was able to detect the general area of the damage, but was not able to locate the damage, and the refined meshing and analysis have to be carried out around the identified areas. The mode shapes of a cable-stayed bridge could be used to indicate the location of the damage but not the extent or intensity of the damage. The acceleration time history response of the Tianjing Yonghe Bridge was used to identify girder or support damage by Liang et al. [8], Huang et al. [9], Bisheh et al. [10,11], and Entezami et al. [12]. To eliminate the ambient temperature influences on the bridge frequency, a damage identification method based on Kalman filter and cointegration (KFC) was developed by Huang et al. [9]. Feature extraction and selection methods were proposed to identify the damage by Bisheh et al. [10,11] and Entezami et al. [12]. Based on acceleration responses and strain responses, a damage identification method was proposed by Alamdari et al. [13]. This method applies incremental tensor analysis for data fusion and feature extraction, and then a one-class support vector machine is used to detect anomalies. Finite element (FE) model updating is a well-recognized approach for SHM purposes, as an accurate model serves as a baseline reference for damage detection and long-term monitoring efforts. The latest advances in finite element modeling and model updating of cable-stayed bridges were presented by Sharry et al. [14]. In addition, influence lines or static methods are also used for damage identification of cable-stayed bridges. The displacement influence line (DIL) of the bridge under live load tests was used to identify the damage of a cable-stayed bridge by Alamdari et al. [15]. This method can identify the damage location, and cannot identify the damage severity.

Furthermore, cables are the crucial components in cable-stayed bridges, which bear the supporting role similar to piers. They are prone to deterioration and damage because of fatigue and corrosion [16,17]. Consequently, the design service life of a cable-stayed bridge is 100 years, while the cable life is generally only 15–20 years in China. If some cables are damaged, the redistribution of cable forces will lead to or accelerate the damage of more cables, which poses a serious threat to the safety, integrity, and static and dynamic characteristics of bridge structures. Therefore, damaged cable identification is important for cable-stayed bridges. Most of the cable monitoring approaches obtain the cable forces through various ways to evaluate the damage state of the cables [18]. A damage assessment and warning method for stay cables based on the acoustic emission (AE) technique and the fractal theory was developed by Li et al. [19]. A hybrid structural health monitoring approach for condition assessment of cable-stayed bridges was presented by Arjomande et al. [20]. The structural integrity of cables is evaluated through incorporating visual inspection, ultrasound test, and local and global vibration analysis data. A combinational identification method of three efficient techniques, including statistical analysis, clustering, and neural network models, was proposed to detect damaged cables in a cable-stayed bridge by Son et al. [21]. A vibration-based model-free damage diagnosis method for stay cables using the changes in natural frequencies was proposed by An et al. [22]. This method divides the stay cable into a short part and a long part by a steel bar. The local frequency change in the short part due to the damage in the whole stay cable is amplified dramatically. Then, small damage of a stay cable can be diagnosed. Based on the vibration signal, a Shannon entropy-based methodology for detecting and locating a lost cable in a cable-stayed bridge exposed to ambient vibrations was presented by Jose et al. [23]. A methodology based on statistical features, principal component analysis (PCA), and Mahalanobis distance (MD) for detecting and locating cable loss using vibration signals was proposed by Jesus et al. [24]. These two methods were validated in the Rio Papaloapan Bridge (Veracruz, Mexico) with a 100% effectiveness to detect the lost cable location. Based on the concept of influence surface, the slope of the linear relationship of the matched cable tension ratio of two cables located on the same side is used as the damage sensitive feature. A long-term condition assessment method for stay cables in cable-stayed bridges using the monitored cable tension forces under operational conditions was developed by Peng et al. [25]. An improved residual force algorithm independent of the static load vector for cable damage identification in cable-stayed bridges was proposed by Fang et al. [26]. By combining two different static loading modes, a damage indicator vector was defined for damage localization and quantification. The relative strain variation in the anchor was used to detect wire breakage in unbonded tendons by Abdullah et al. [27]. The feasibility of an impedance-based stress monitoring method for local-strand breakage detection in multi-strand anchorage systems was investigated by Dang et al. [28]. A method to detect the location and the magnitude of the damaged cables of cable-stayed bridges based on the dynamic distributed sensing of bridge deck strains was introduced by Scarella et al. [29].

All the above studies identified the damage on girders or cables in cable-stayed bridges. However, there are still some limitations. For instance, most of the methods can only identify high-severity damage, and have a low anti-noise capability. Furthermore, few of them could identify multiple damage. To overcome the above limitations, the authors are committed to solving these problems, and a support vector machine (SVM)-based method was developed to identify single and double damaged cables from bridge deck strain differences in a previous study [18]. The data from all damage scenarios are needed to train the SVM model for damage detection. However, it is difficult or even impossible to obtain the data for all damage scenarios, especially multiple damage scenarios. Therefore, this paper adopts the single damaged cable identification index vector in the previous paper [18] to construct a basis vector matrix (BVM), and a new method based on the BVM is proposed for identification of multiple damaged cables. This method can directly identify single or multiple damaged cables, including early small damage. The relationship between the cable cross-section area and the bending strain of the bridge deck is established firstly using a force method. Then, the damage index vector (DIXV) is obtained from the difference between the maximum bending strains of the bridge deck with intact and damaged cables. The DIXV is normalized in [0, 1] to obtain the BVM. Finally, the damage indicator vector (DIV) is obtained by the relationship between the DIXV and BVM to identify the damaged cables. The proposed method is verified using a field cable-stayed bridge for identification of single or multiple damaged cables.

## 2. Basis Vector Matrix Method

### 2.1. Relationship between the Cable Tension and the Bending Strain

**Figure 1:**
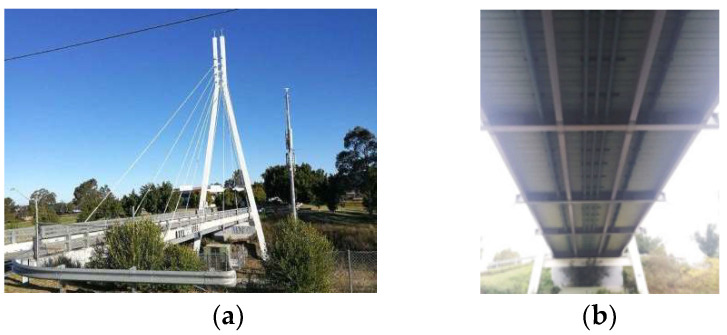
A single tower cable-stayed bridge. (**a**) The cable-stayed bridge; (**b**) the girder layout [1].

**Figure 2:**
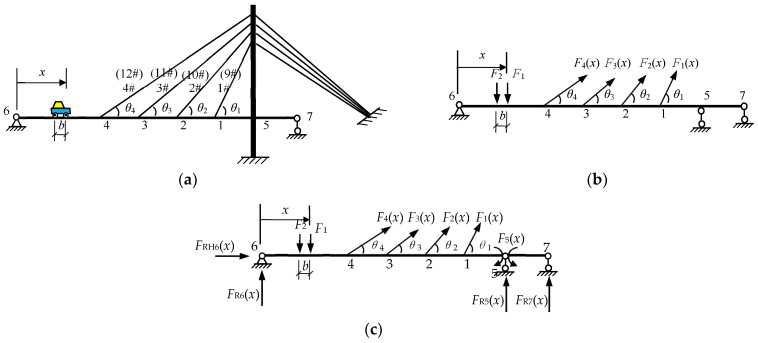
The cable force calculation diagram of the cable-stayed bridge. (**a**) A simple plane calculation diagram; (**b**) calculation diagram after the cables substituted by the cable forces; (**c**) the force method basic system.

The damage of a cable is mainly caused by the fracture or failure of the steel wire due to corrosion, fatigue, or overload, and it results in a decrease in the effective cross-section area of the cable. Therefore, the damage of the cable is usually simulated by reducing the cross-sectional area of the cable in the finite element model. At the same time, when the cable is damaged, the bearing capacity of the cable will be decreased and the cable forces of the whole bridge will be redistributed. Then, the internal force and strain of the bridge are changed correspondingly. To illustrate the relationship between the cable damage and the bending strain of the girder underneath, a single tower cable-stayed bridge is used, as shown in Figure 1. The side view of the cable-stayed bridge is shown in Figure 2a. The left end of the bridge is a pin support to restrict the vertical and longitudinal linear displacements, and the right end and the tower are roller supports to restrict the vertical linear displacement, as shown in Figure 2b. The vehicle is considered as two moving concentrated forces *F*_1_ and *F*_2_. In Figure 2a, the beam bridge is a statically indeterminate continuous beam. The force method can be used to calculate the internal force of the cable-stayed bridge under the vehicle at *x*, and the relationship between the cable tensile stiffness *EA* and the bending strain of the beam can be derived. The derivation procedure is as below.

The basic system is obtained by removing redundant constraints, as shown in Figure 2c. The equation is established by the force method,

(1) $$\left\{\begin{array}{c} \Delta_{1}\left(x\right)\\ \Delta_{2}\left(x\right)\\ \Delta_{3}\left(x\right)\\ \Delta_{4}\left(x\right)\\ \Delta_{5}\left(x\right) \end{array}\right\}=\left[\begin{array}{ccccc} \delta_{11} & \delta_{12} & \delta_{13} & \delta_{14} & \delta_{15}\\ \delta_{21} & \delta_{22} & \delta_{23} & \delta_{24} & \delta_{25}\\ \delta_{31} & \delta_{32} & \delta_{33} & \delta_{34} & \delta_{35}\\ \delta_{41} & \delta_{42} & \delta_{43} & \delta_{44} & \delta_{45}\\ \delta_{51} & \delta_{52} & \delta_{53} & \delta_{54} & \delta_{55} \end{array}\right]\left\{\begin{array}{c} F_{1}\left(x\right)\\ F_{2}\left(x\right)\\ F_{3}\left(x\right)\\ F_{4}\left(x\right)\\ F_{5}\left(x\right) \end{array}\right\}+\left\{\begin{array}{c} \Delta_{1P}\left(x\right)\\ \Delta_{2P}\left(x\right)\\ \Delta_{3P}\left(x\right)\\ \Delta_{4P}\left(x\right)\\ \Delta_{5P}\left(x\right) \end{array}\right\}=\left\{\begin{array}{c} -\frac{F_{1}\left(x\right)L_{1}}{E_{1}A_{1}}\\ -\frac{F_{2}\left(x\right)L_{2}}{E_{2}A_{2}}\\ -\frac{F_{3}\left(x\right)L_{3}}{E_{3}A_{3}}\\ -\frac{F_{4}\left(x\right)L_{4}}{E_{4}A_{4}}\\ 0 \end{array}\right\}$$

where $F_{i}\left(x\right)$ is the unknown redundant reaction forces of the redundant constraints. $\Delta_{i}\left(x\right)$ are the displacements corresponding to the unknown redundant reaction forces in the original structure (Figure 2a). $\Delta_{iP}\left(x\right)$ are the displacements corresponding to the unknown redundant reaction forces in the basic structure under the vehicle loads, also called free term. $E_{i},A_{i},L_{i}$ are the *i*th cable’s elastic modulus, cross-sectional area, and length, respectively. $\delta_{ij}$ is the flexibility coefficient, that is, the displacement along the *i*th unknown redundant force when the *j*th unknown redundant force is 1 and the other unknown redundant forces are all 0. Equation (1) can also be rewritten as

(2) $$\left[\begin{array}{c} \delta_{11}+\frac{L_{1}}{E_{1}A_{1}}\\ \delta_{21}\\ \delta_{31}\\ \delta_{41}\\ \delta_{51} \end{array}\begin{array}{c} \delta_{12}\\ \delta_{22}+\frac{L_{2}}{E_{2}A_{2}}\\ \delta_{32}\\ \delta_{42}\\ \delta_{52} \end{array}\begin{array}{c} \delta_{13}\\ \delta_{23}\\ \delta_{33}+\frac{L_{3}}{E_{3}A_{3}}\\ \delta_{43}\\ \delta_{53} \end{array}\begin{array}{c} \delta_{14}\\ \delta_{24}\\ \delta_{34}\\ \delta_{44}+\frac{L_{4}}{E_{4}A_{4}}\\ \delta_{54} \end{array}\begin{array}{c} \delta_{15}\\ \delta_{25}\\ \delta_{35}\\ \delta_{45}\\ \delta_{55} \end{array}\right]\left\{\begin{array}{c} F_{1}\left(x\right)\\ F_{2}\left(x\right)\\ F_{3}\left(x\right)\\ F_{4}\left(x\right)\\ F_{5}\left(x\right) \end{array}\right\}=-\left\{\begin{array}{c} \Delta_{1P}\left(x\right)\\ \Delta_{2P}\left(x\right)\\ \Delta_{3P}\left(x\right)\\ \Delta_{4P}\left(x\right)\\ \Delta_{5P}\left(x\right) \end{array}\right\}$$

Equation (2) can be written as the matrix form,

(3) $$\delta F=-\Delta_{p}$$

where $\delta =\delta_{b}+\delta_{c}$, $\delta_{b}$, and $\delta_{c}$ are the flexibility matrixes, which are respectively related to the parameters of the beam and the cable, independent of the external force. They are written as

(4) $$\delta_{b}=\left[\begin{array}{ccccc} \delta_{11} & \delta_{12} & \delta_{13} & \delta_{14} & \delta_{15}\\ \delta_{21} & \delta_{22} & \delta_{23} & \delta_{24} & \delta_{25}\\ \delta_{31} & \delta_{32} & \delta_{33} & \delta_{34} & \delta_{35}\\ \delta_{41} & \delta_{42} & \delta_{43} & \delta_{44} & \delta_{45}\\ \delta_{51} & \delta_{52} & \delta_{53} & \delta_{54} & \delta_{55} \end{array}\right]$$

(5) $$\delta_{c}=\left[\begin{array}{c} \frac{L_{1}}{E_{1}A_{1}}\\ 0\\ 0\\ 0\\ 0 \end{array}\begin{array}{c} 0\\ \frac{L_{2}}{E_{2}A_{2}}\\ 0\\ 0\\ 0 \end{array}\begin{array}{c} 0\\ 0\\ \frac{L_{3}}{E_{3}A_{3}}\\ 0\\ 0 \end{array}\begin{array}{c} 0\\ 0\\ 0\\ \frac{L_{4}}{E_{4}A_{4}}\\ 0 \end{array}\begin{array}{c} 0\\ 0\\ 0\\ 0\\ 0 \end{array}\right]$$

***F*** is the column vector of the unknown redundant reaction forces. $\Delta_{p}$ is the column vector of the free terms.

From Equation (3), ***F*** is obtained as

(6) $$F=-\delta^{-1}\Delta_{p}$$

Then, the redundant reaction forces F can be regarded as the external loads acting on the basic structure shown in Figure 2c. When the basic structure is statically determinate, the bending moments $M\left(x^{\prime }\right)$ can be obtained at any cross-sections by the static equilibrium equation. Further, the bending strain $\varepsilon \left(x^{\prime }\right)$ at the lower beam of any cross-section $x^{\prime }$ can be obtained as

(7) $$\varepsilon \left(x^{\prime }\right)=\frac{M\left(x^{\prime }\right)y}{EI}$$

where $M\left(x^{\prime }\right)$ is the bending moment at the cross-section $x^{\prime }$, *y* is the distance between the neutral axis and the bottom surface of the cross-section $x^{\prime }$, and *EI* is the bending stiffness of the beam.

From Equations (2), (6) and (7), when the cable cross-section area is reduced, the cable forces will be redistributed. As a result, the bending moments and bending strains on the beam also change accordingly. It means that the change in the bending strain of the beam can reflect the damage of the cable. Similarly, the equations for the three-dimensional bridge structure under vehicle loads can be obtained but that is not in the scope of this paper.

### 2.2. Damage Index for the Damaged Cables of Cable-Stayed Bridges

It is well known that damage will reduce the tensile stiffness *EA* of the cable. In order to facilitate simulation, it is assumed that the elastic modulus *E* is constant when the cable is damaged. Then, the cable damage is described using the cable cross-section area reduction. The cable damage severity is defined as

(8) $$de=\frac{EA_{\mathrm{in}}-EA_{\mathrm{da}}}{EA_{\mathrm{in}}}\times 100\%=\frac{A_{\mathrm{in}}-A_{\mathrm{da}}}{A_{\mathrm{in}}}\times 100\%$$

where *de* is the cable damage severity, and *A*_in_ and *A*_da_ are the cross-section areas of the intact and damaged cables, respectively.

In practice, the bridge deck bending strains under operational conditions can be monitored through a long-term monitoring system. When a vehicle passes over the bridge, the bending strains are varied over time and that is related to the vehicle location on the bridge. In this study, the maximum bending strains of the time history strain responses at measurement locations are extracted as the features of the bending strain data. The measurement locations on the bridge deck are around the cable anchors [18]. Then, the damage index vector (DIXV) is defined as the absolute value of the difference between the maximum bending strains of the bridge at measurement points with or without damaged cables, that is,

(9) $$DIXV^{j\#,de}={\left(x_{1}^{j\#,de}\ldots x_{i}^{j\#,de}\ldots x_{n}^{j\#,de}\right)}^{T}={\left(\left|\Delta \varepsilon_{1}\right|\ldots \left|\Delta \varepsilon_{i}\right|\ldots \left|\Delta \varepsilon_{n}\right|\right)}^{T}$$

where $DIXV^{j\#,de}$ and $x_{i}^{j\#,de}$ are DIXV and the *i*th damage index for cables of the bridge with the *j*# damaged cable. n is the total number of measuring points. $\left|\Delta \varepsilon_{i}\right|\;$ is

(10) $$\left|\Delta \varepsilon_{i}\right|=\left|\max\left(\varepsilon_{i\mathrm{in}}\right)-\max\left(\varepsilon_{i\mathrm{da}}\right)\right|$$

where $\max\left(\varepsilon_{i\mathrm{in}}\right)$ and $\max\left(\varepsilon_{i\mathrm{da}}\right)$ are the maximum bending strains of the *i*th measured point with or without the damaged cable, respectively.

### 2.3. Basis Vector Matrix

When the *j*# cable damage severity is de, $DIXV^{j\#,de}$ is normalized as

(11) $$X_{i}^{j\#}=\frac{x_{i}^{j\#,de}}{x_{\max}^{j\#,de}}$$

where $x_{\max}^{j\#,de}$ is the maximum value of $DIXV^{j\#,de}$, and $X_{i}^{j\#}$ is the corresponding normalized value of the *i*th measurement point with the *j*# damaged cable.

Then, the basis vector $BV^{j\#}$ with the *j*# damaged cable is obtained.

(12) $$BV^{j\#}={\left(X_{1}^{j\#}\cdots X_{i}^{j\#}\ldots X_{n}^{j\#}\right)}^{T}$$

The basis vector matrix (***BVM***) can be written as

(13) $$BVM=\left[BV^{1\#}BV^{2\#}\cdots BV^{j\#}\cdots BV^{m\#}\right]={\left[\begin{array}{cc} \begin{array}{ccc} X_{1}^{1\#} & X_{1}^{2\#} & \cdots \\ \vdots & \vdots & \cdots \\ X_{i}^{1\#} & X_{i}^{2\#} & \cdots \end{array} & \begin{array}{ccc} X_{1}^{j\#} & \cdots & X_{1}^{m\#}\\ \vdots & \vdots & \vdots \\ X_{i}^{j\#} & \cdots & X_{i}^{m\#} \end{array}\\ \begin{array}{ccc} \vdots & \vdots & \cdots \\ X_{n}^{1\#} & X_{n}^{2\#} & \cdots \end{array} & \begin{array}{ccc} \vdots & \vdots & \vdots \\ X_{n}^{j\#} & \cdots & X_{n}^{m\#} \end{array} \end{array}\right]}_{n\times m}$$

where ***BVM*** is the basis vector matrix, *m* is the number of damaged cables. ***BVM*** is an *n* × *m* matrix.

### 2.4. Damaged Cable Identification

From Equations (11)–(13), $DIXV^{j\#,de}$ can be expressed as

(14) $$DIXV^{j\#,de}=BVM\cdot DIV=BV^{j\#}\cdot x_{\max}^{j\#,de}$$

where ***DIV*** is the damage indication vector (DIV),

(15) $$DIV={\left(0\cdots x_{max}^{j\#,de}\ldots 0\right)}^{T}$$

From Equation (14), ***DIV*** can be calculated as below:

(16) $$DIV=BVM^{-1}\cdot DIXV^{j\#,de}$$

where $BVM^{-1}$ is the inverse matrix of BVM. In fact, for the case with the *j*# damaged cable, all components of ***DIV*** should be 0, except the *j*th component is $x_{\max}^{j\#}\neq 0$. According to the location of the nonzero components in ***DIV***, the number of damaged cables can be identified. For the scenario with several damaged cables, the location of the nonzero components in ***DIV*** correspond to the number of damaged cables. In practice, there may be some small nonzero items in the ***DIV*** due to noise interference, and a threshold can be set to eliminate the noise effect. For example, the threshold can be determined by the maximum value in the ***DIXV*** that corresponds to the minimum allowable damage severity, such as 3%, etc.

After the damaged cables are identified, the damaged severity of these cables can be identified in the next step. It is known that the maximum value $\;x_{\max}^{j\#}$ in $DIXV^{j\#}$ increases with the increase in damage severity *de* [18]. When the cable damage severity *de* varies continuously, $x_{\max}^{j\#}$ is a nonlinear function of *de.* The relationship between $x_{\max}^{j\#}$ and *de* can be established using the nonlinear regression method, that is,

(17) $$de_{j}=f_{j}\left(x_{\max}^{j\#}\right)$$

where $de_{j}$ is the damage severity of the *j*# cable, *f_j_*( ) is the nonlinear functional relationship between $de_{j}$ and $x_{\max}^{j\#}$, that is, the damage severity identification function.

Finally, the damage severity of the *j*# cable can identified by substituting the nonzero component $DIV_{j}$ into Equation (17).

**Figure 3:**
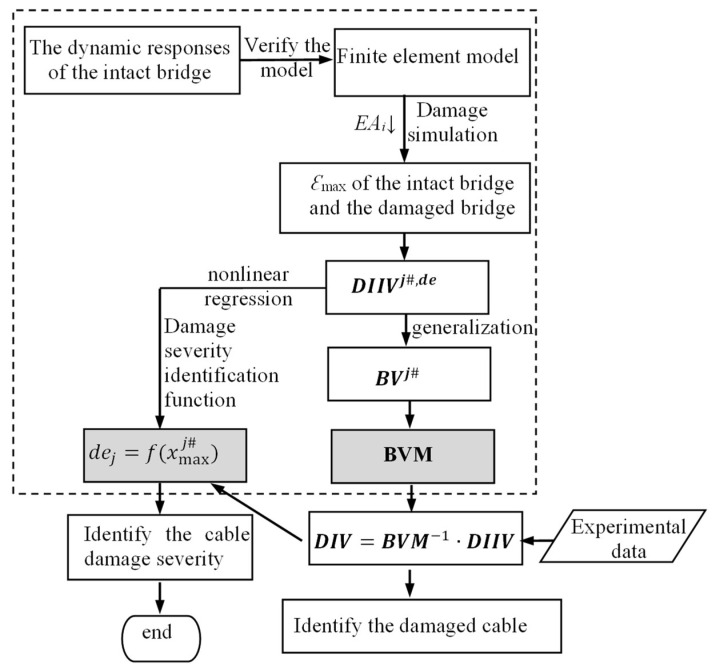
The flow chart of the BVM damaged cable identification procedure.

The flow chart of the ***BVM*** method for the damaged cable identification is shown in Figure 3.

## 3. Applications

### 3.1. Description of the Structural Health Monitoring System

**Figure 4:**
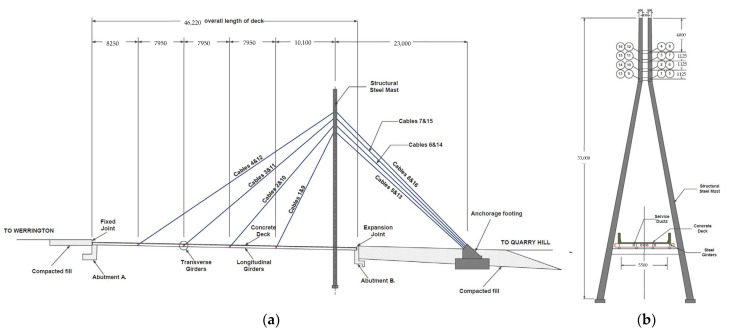
Schematic view of the cable-stayed bridge [1]. (**a**) Elevation view of the bridge; (**b**) bridge mast.

**Figure 5:**
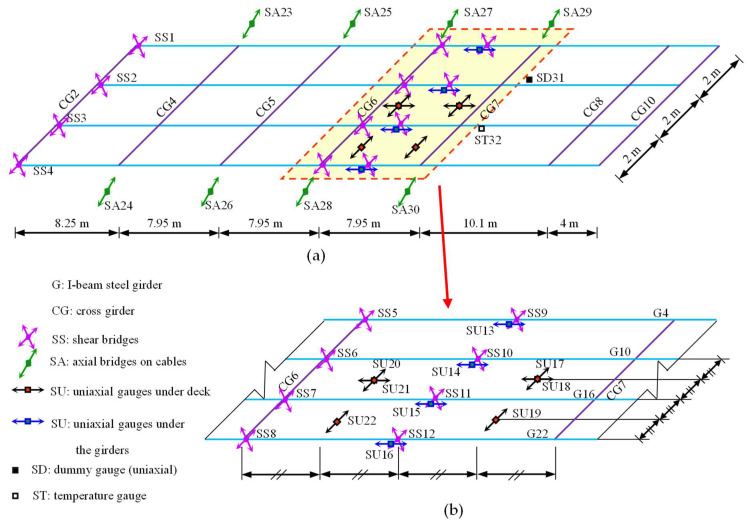
Illustration of the strain gauge array of the cable-stayed bridge structural health monitoring system. (**a**) The deck plan; (**b**) the strain gauge array between CG6 and CG7.

**Figure 6:**
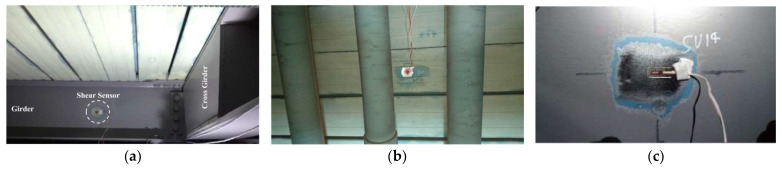
Sensor location on the cable-stayed bridge [30]. (**a**) Shear strain gauge on the web of the girder; (**b**) uniaxial gauge under the deck; (**c**) uniaxial gauge under the flange of the girder.

A field cable-stayed bridge, as shown in Figure 1 and Figure 4, is used to verify the proposed method. The bridge is a single-lane highway bridge with a span of 46 m and a width of 6.3 m. There are 16 stay cables in a semi-fan arrangement and the single A-shaped steel tower is 33 m high. The bridge deck is a composite steel–concrete deck. The concrete deck has a thickness of 0.16 m, it is supported by four I-beam steel girders. The girders are internally attached by a set of equally spaced cross-girders, shown in Figure 1b [1]. A long- term monitoring system has been installed on the bridge with an array of strain gauge sensors installed under the bridge deck at the intersection of the girders and floor beams (shown in Figure 5). Figure 5b shows the magnified view of the strain gauge array between CG6 and CG7 marked in the yellow area in Figure 5a. Figure 6 shows the sensor locations of the shear strain gauge and the uniaxial strain gauges on the cable-stayed bridge. An HBM Quantum-X data acquisition system (HBM, Darmstadt, Germany) was adopted for signal conditioning and data logging. The Quantum system provides an integrated and reliable device to log high-quality data with 24-bit resolution with a bandwidth capability of 0–3 kHz. The response signals of the bridge were collected at 600 Hz while test vehicles were traveling over the bridge [30].

### 3.2. Finite Element Model of the Cable-Stayed Bridge

**Figure 7:**
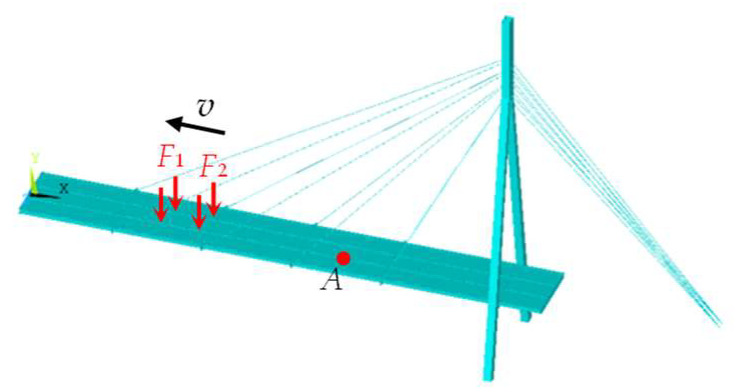
The FEM of the cable-stayed bridge under vehicle loads [18].

Figure 7 shows the finite element model (FEM) of the cable-stayed bridge which is the same as the FEM in the literature [18]. The steel-reinforced concrete part is simulated by SHELL63 with a thickness of 160 mm. The lower longitudinal and transverse girders are simulated by BEAM189 with Universal Beam (410UB54) cross-section properties. The cables are simulated by LINK10. The bridge mast is simulated by BEAM189 with variable cross-sections, and all degrees of freedom of the mast base are restrained. As shown in Figure 1 and Figure 2, the left end of the bridge is a pin support under every longitudinal girder and the right end of the bridge is a roller support. The vehicle load is simulated by four moving concentrated forces acting on the bridge.

**Figure 8:**
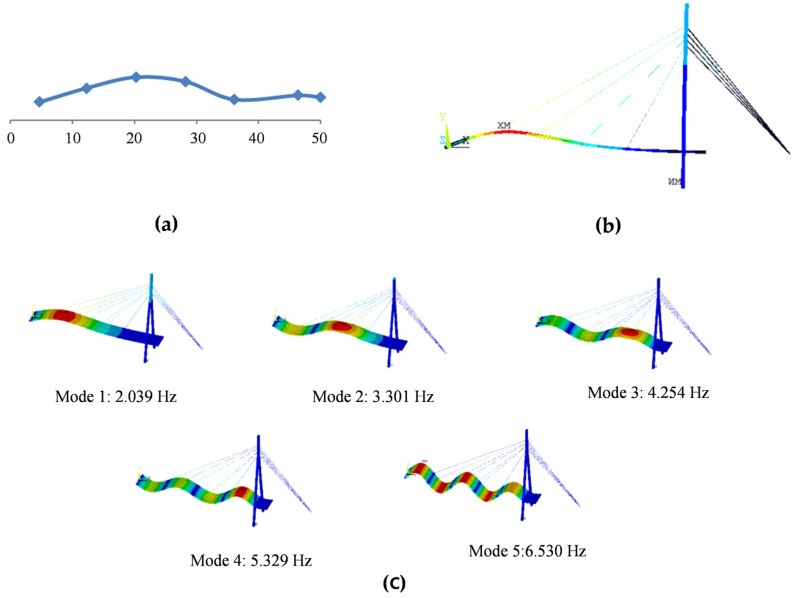
The first five mode shapes of the cable-stayed bridge [18]. (**a**) The 1st test mode shape [32]; (**b**) the 1st FEM mode shape at side; (**c**) the first five FEM mode shapes in 3D perspective.

To verify the FEM of the cable-stayed bridge, the frequencies and modes are calculated using FEM and compared with the field testing results. Table 1 lists the first five frequencies of the cable-stayed bridge by FEM and the comparison with the field testing results by Sun et al. [31]. Figure 8 shows the comparison of the first mode shapes by FEM and the field testing [32].

In Table 1, the mode’s modal assurance criterion (MAC) [33] is calculated by

(18) $$MAC_{i}=\frac{{\left|\emptyset_{i}^{c}{}^{T}\emptyset_{i}^{m}\right|}^{2}}{\left(\emptyset_{i}^{c}{}^{T}\emptyset_{i}^{c}\right)\left(\emptyset_{i}^{m}{}^{T}\emptyset_{i}^{m}\right)}$$

where $\emptyset_{i}^{c}$ and $\emptyset_{i}^{m}$ are the ith calculated modal vector and measured modal vector, respectively. When MACi is closer to 1, $\emptyset_{i}^{c}$ agrees well with $\emptyset_{i}^{m}$.

From Table 1, the frequencies by FEM are very close to the experimental results, and the maximum difference is 12.154%, which is the third frequency. Especially, the difference between the first frequencies by FEM and the test is 1.192%, and the corresponding *MAC*_1_ is 0.978, close to 1. It should be mentioned that only the first mode is measured and only the *MAC* of the first mode is calculated in Table 1.

In Figure 8, it can also be found that the 1st modal shapes by FEM and the test agree well. Therefore, the FEM of the cable-stayed bridge can represent the bridge.

**Figure 9:**
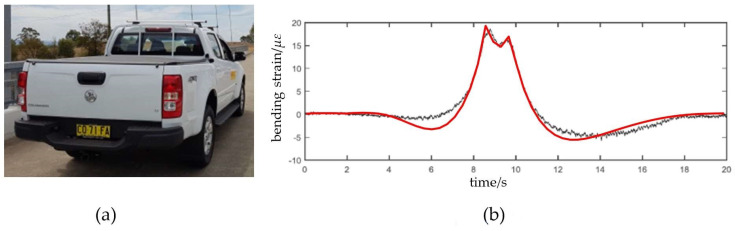
The test vehicle and the bending strains. (**a**) Holden Colorado Ute [1]; (**b**) the measured bending strain (black line) [1] and the FEM bending strain (red line) [18].

Further, as ***DIXV*** is calculated using the bending strains, the bending strains by FEM are also compared with the measured data to further verify the model. The measurements of sensor SU15 (shown in Figure 5), which is located at point A in Figure 7, are used. The field test vehicle is a Holden Colorado Ute, as shown in Figure 9a. The gross weight of the test vehicle is 2.20 t, with front and rear axle loads of *F*_1_ = 1.20 t and *F*_2_ = 1.00 t, respectively. The distance between these two axles is 3.10 m, and the wheel spacing is 1.75 m [1]. The axle load is evenly distributed between two wheels. Therefore, the vehicle load is simplified as four moving concentrated forces acting on the bridge, shown in Figure 7. The test vehicle passed the bridge at a constant speed of v = 10 km/h along the center line of the bridge deck. Figure 9b shows the comparison of the bending strains of the longitudinal girder at point *A* by FEM and the measurements. From Figure 9b, the results show that they have a similar trend, and the bending strain peaks corresponding to the front and rear wheels, respectively, are very close. It shows that the proposed model is reliable and accurate to determine the bending strains. By Equations (9) and (10), ***DIXV*** can be calculated by the maximum bending strains. Therefore, the finite element model is reliable and accurate to obtain the basis vector matrix ***BVM***.

### 3.3. Cable Damage Identification

**Figure 10:**
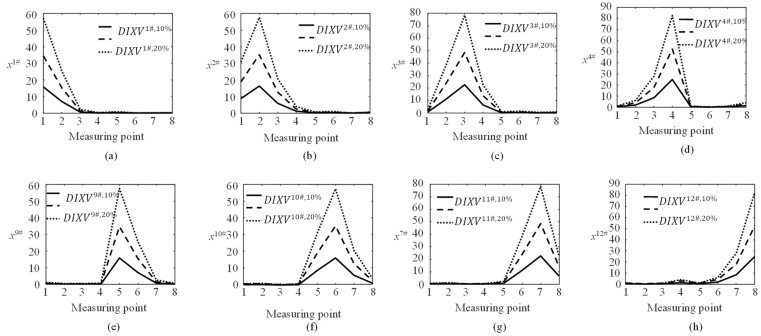
The poly lines of DIXV with 10%, 20%, and 30% in Cables 1#–4# and Cables 9#–12#. (**a**) Cable 1#; (**b**) Cable 2#; (**c**) Cable 3#; (**d**) Cable 4#; (**e**) Cable 9#; (**f**) Cable 10#; (**g**) Cable 11#; (**h**) Cable 12#.

As shown in Figure 2a, the cables are symmetrically arranged along the longitudinal center line of the bridge deck, and they are numbered as listed in the brackets. Since Cables 5#–8# and Cables 13#–16# are directly anchored on the anchorage footing, only the damage identification of Cables 1#–4# and Cables 9#–12# is studied here. The damage is simulated in Cables 1#–4# and Cables 9#–12# with damage severities of 10%, 20%, and 30%, respectively. ***DIXV***s are calculated and their poly line diagrams are shown in Figure 10. The measured points are on the bottom surface of the longitudinal girders close the anchors of Cables 1#–4# and Cables 9#–12#.

Figure 10 shows that the $DIXV^{j\#,de}$ poly lines are significantly different from $DIXV^{k\#,de}$ for *j* ≠ *k*. When Cable *j*# is damaged, the maximum value of $DIXV^{j\#,de}$ increases with the damage severity. For two cables in symmetrical positions, such as Cables 1# and 9#, the value of ***DIXV*** at measured position 1 for Cable 1#, due to its damage, is equal to that at the symmetrical measurement point 5 due to the damage in Cable 9#.

**Figure 11:**
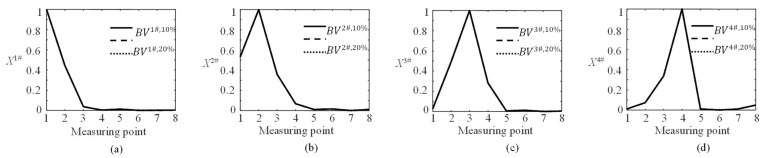
The poly line diagrams of $BV^{j\#,de}$ for Cables 1#–4# with 10%, 20%, and 30% damage, respectively. (**a**) Cable 1#; (**b**) Cable 2#; (**c**) Cable 3#; (**d**) Cable 4#.

The ***DIXV***s are normalized to obtain the basic vectors $BV^{j\#,de}$ by Equation (11). Figure 11 shows the poly line diagrams of the normalized ***DIXV***s for Cables 1#–4#, and they are $BV^{j\#,de}$. The diagrams for Cables 9#–12# can be obtained by the symmetry.

As shown in Figure 11, the basis vectors are not changed with the damage severity. For simplification, $BV^{j\#,de}$ can be abbreviated as $BV^{j\#}$. For Cable *j*#, $BV^{j\#}$ can be obtained from the corresponding $DIIV^{j\#,de}$. Then, the basic vector matrix (BVM) for damaged cable identification can be written as

(19) $$BVM=\left[BV^{1\#}\;BV^{2\#}\;BV^{3\#}\;BV^{4\#}\;BV^{9\#}\;BV^{10\#}\;BV^{11\#}\;BV^{12\#}\right]$$

**Figure 12:**
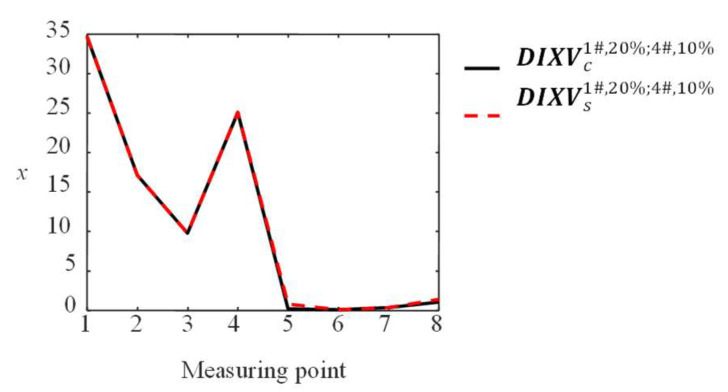
Comparison of $DIIV_{c}^{1\#,20\%;4\#,10\%}$ obtained by FEM and $DIIV_{s}^{1\#,20\%;4\#,10\%}$ obtained by superposition.

The DIXV for two damage cables is also studied here. $DIXV_{c}^{1\#,20\%;4\#,10\%}$ is the ***DIXV*** using FEM when Cables 1# and 4# have 20% and 10% damage, respectively. $DIXV_{s}^{1\#,20\%;4\#,10\%}$ is obtained by the sum of $DIXV^{1\#,20\%}$ and $DIXV^{4\#,10\%}$, e.g., $DIXV_{s}^{1\#,20\%;4\#,10\%}=DIXV^{1\#,20\%}+DIXV^{4\#,10\%}$. Their poly line diagrams are shown in Figure 12.

Figure 12 shows that the two poly lines are very close to each other. It means that $DIIV_{c}^{1\#,20\%;4\#,10\%}$ is almost equal to $DIIV_{s}^{1\#,20\%;4\#,10\%}$, and the proposed BVM method in Section 2 can be used to identify multiple damaged cables simultaneously.

### 3.4. The Hypothetical Damage Scenarios

To verify the performance and robustness of the proposed method, the hypothetical damage scenarios are listed in Table 2, including single-damage scenarios and multiple-damage scenarios. ***DIXV***s for all damage scenarios are calculated by FEM and white noise is added to simulate the measurements by Equation (20).

(20) $$DIXV^{j,\varepsilon }=DIXV\times \left(1+\varepsilon R_{i}\right)\left(i=1,2,\cdots ,m\right)$$

where $DIXV^{j,\varepsilon }$ is the DIXV of the *j*th damaged scenario with the measurement noise level *ε*. Here, *ε* is 5%, 10%, 15%, and 20%, respectively. $R_{i}$ is the *i*th value of the normally distributed random data with a mean value of 0 and a standard deviation of 1. *m* is total number of measured points. Eight measurement points corresponding to Cables 1#–4# and Cables 9#–12# are adopted.

To verify the robustness of the proposed method, 100 samples for the DIXV of each damage scenario are generated by adding the noise. For instance, $DIXV_{k}{}^{1\#,5\%}$ has 100 DIXVs with 5% measurement noise, k = 1,2,⋯,100. The test dataset with 2800 DIXVs is obtained.

Here, BVM is only constructed by $BV^{j\#,20\%}$, and is used to identify the damaged cables with other damage severities, such as 10%, 30%, to test the performance of the proposed BVM method.

### 3.5. Damaged Cable Identification

**Figure 13:**
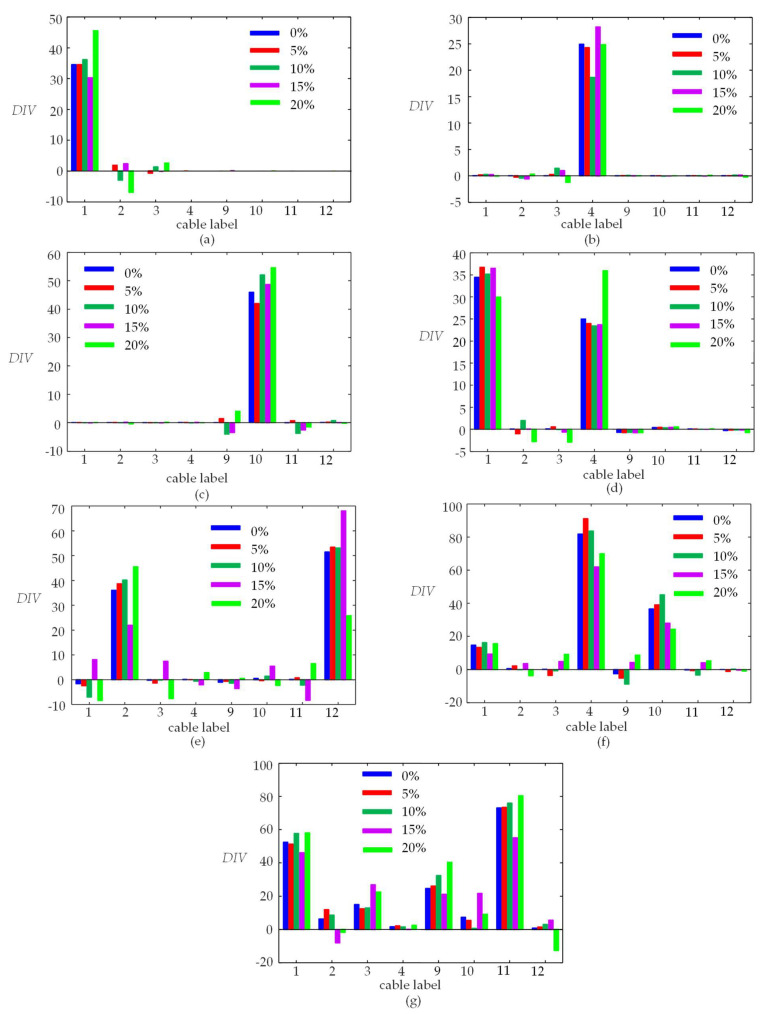
Histogram of DIV obtained from a set of DIXV for each damage scenario without noise and adding 5%, 10%, 15%, and 20% noise, respectively. (**a**) Scenario ①; (**b**) Scenario ②; (**c**) Scenario ③; (**d**) Scenario ④; (**e**) Scenario ⑤; (**f**) Scenario ⑥; (**g**) Scenario ⑦.

In this section, the BVM method is used to identify the damaged cable labels in Table 2. The test dataset is substituted into Equation (16) to obtain DIV. The components in DIV are compared with the set threshold to identify the damaged cable labels and the threshold is 5 in this study, which is determined by the maximum value in $DIXV^{1\#,3\%}$. Figure 13 shows the DIV histogram for seven damage scenarios calculated by the DIXV with different noise levels. Table 3 lists the identified damaged cables using the BVM method and the SVM method [18], respectively.

From Figure 13, the DIV values corresponding to the damaged cables are much larger than that of the intact cables. Although the measurement noise has a large influence on DIV, the components corresponding the damaged cables are still identified successfully. With the noise level increases and more cables damaged simultaneously, some components corresponding to intact cables may be larger than the threshold value 5.

From Table 3, the SVM method [18] can only correctly identify the damaged cables for Scenarios ①~⑤, which are single or double cable damage scenarios. The BVM method can correctly identify the damaged cables in Scenarios ①~④ regardless of noise level. For Scenarios ⑤ and ⑥, the damaged cables are still identified successfully when the noise level is under 15%. With the noise level increasing, some intact cables adjacent to the damaged cables are misidentified. For the four damaged cables in Scenario ⑦, the damaged cables are correctly identified and the cables adjacent to damaged ones are misidentified.

**Figure 14:**
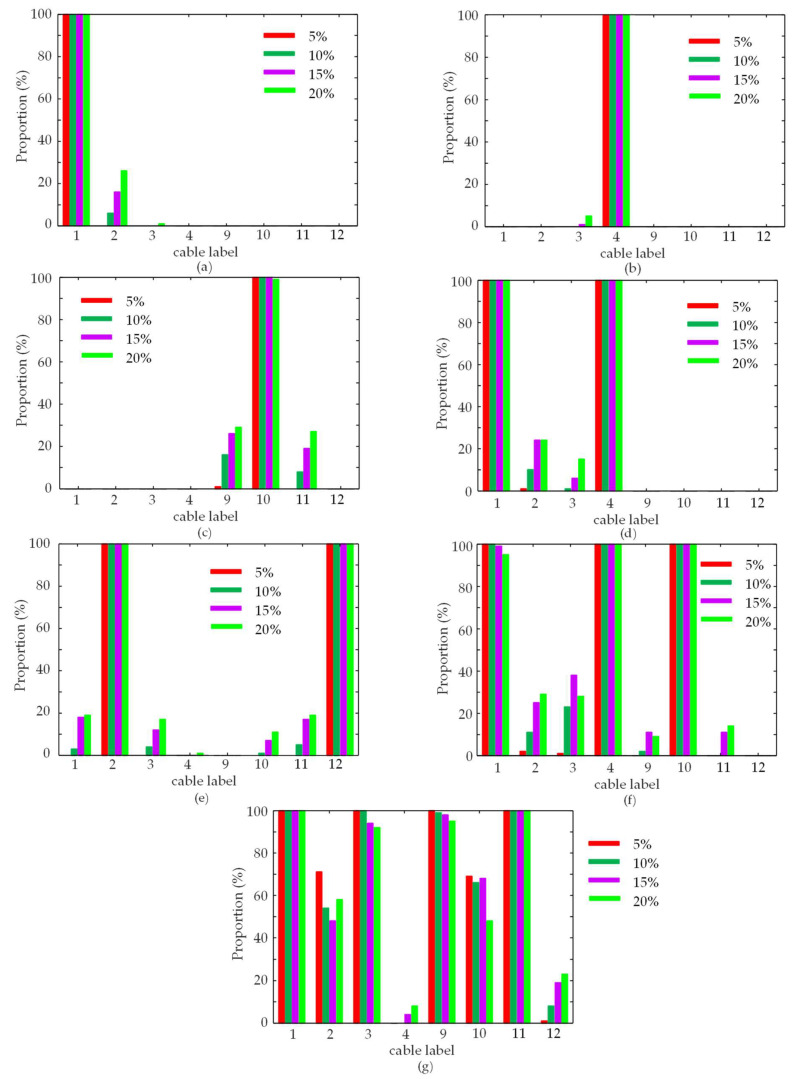
The sample proportion histogram of the cable labels identified by the test dataset with 2800 DIXVs. (**a**) Scenario ①; (**b**) Scenario ②; (**c**) Scenario ③; (**d**) Scenario ④; (**e**) Scenario ⑤; (**f**) Scenario ⑥; (**g**) Scenario ⑦.

To deal with the misidentified cables, Figure 14 shows the sample proportion histogram of the identified cables from the test dataset with 2800 DIXVs. From Figure 14, it can be seen that the correct sample proportions of the damaged cables are all above 90%. For Scenarios ①~⑤, the sample proportions are approximately 100%. Although the sample proportion of Cable 3# is the lowest among the damaged cables in Scenario ⑦, it is 92%. For other damage scenarios, the sample proportion of any intact cables is less than 40%. For Scenario ⑦ with four damaged cables, the sample proportion of the intact Cable 2# is only 71%.

The proportion confidence interval is calculated by Equation (21) to validate this method’s reliability.

(21) $$\left(p-z\sqrt{\frac{p\left(1-p\right)}{n}},p+z\sqrt{\frac{p\left(1-p\right)}{n}}\;\right)$$

where *p* is the sample proportion, *z* is the critical value corresponding to the confidence level, *n* is the sample size. Here, the confidence level is 95%, the corresponding *z* is 1.96, and *n* is 100.

For the lowest sample proportion of 92% of the damaged Cable 3# in Scenario ⑦, the proportion confidence interval is (87%, 97%). For the highest sample proportion of 71% of the intact Cable 2# in Scenario ⑦, the proportion confidence interval is (62%, 80%). Therefore, in actual cable-stayed bridge damage identification, it is recommended to collect several sets of DIXV as far as possible to identify the damaged cables. When the sample identification proportion of a cable is greater than 90%, it can be confirmed as a damaged cable.

In summary, the BVM method proposed in this paper can quickly and accurately identify single damaged cable and multiple damaged cable scenarios at one time. It also has a good generalization and anti-noise capability.

### 3.6. Damage Severity Identification

The damage scenarios in Table 2 are used in this section. Table 4 shows the damage severity identification functions. These functions are obtained by cubic polynomial regression in Matlab based on the maximum values of DIXV when the cables have 10%, 20%, and 30% damage, respectively. Here, x is the nonzero component in DIV, which is greater than the threshold value.

The damage severity can be identified by substituting the component x into the corresponding cable damage severity identification function. Table 5 and Table 6, respectively, list the identified results and errors for seven damage scenarios corresponding to Figure 13 using the BVM method and the SVM method [18]. The error is calculated by the difference between the identified damage severity and the true damage severity as

(22) $$error_{i}=de_{i}-D_{i}$$

where *de_i_* and *D_i_* are the identified and true damage severities of the *i*th cable.

The performance of the BVM method and the SVM method [18] is measured by mean squared error (*MSE*) [34], and the square of the regression correlation coefficient (*R*^2^) and the uncertainty interval (U95) [35]. The mathematical relations of these parameters are given as

(23) $$MSE=\frac{1}{N}\sum_{i=1}^{N}{\left(de_{i}-D_{i}\right)}^{2}$$

(24) $$R^{2}=\frac{\sum_{i=1}^{N}de_{i}{}^{2}}{\sum_{i=1}^{N}de_{i}{}^{2}-\sum_{i=1}^{N}{\left(de_{i}-D_{i}\right)}^{2}}$$

(25) $$U95=\frac{1.96}{N}\sqrt{\sum_{i=1}^{N}{\left(D_{i}-\bar{D}\right)}^{2}+\sum_{i=1}^{N}{\left(D_{i}-de_{i}\right)}^{2}}$$

where *N* is the number of data samples, and here it is 100. $\bar{D}$ is the mean value of the true damage severity, and here it is *D_i_*. When *MSE* is closer to 0, *R*^2^ is closer to 1, and U95 is closer to 0, the performance of this method is better. The *MSE*, *R*^2^, and U95 of the identified results for seven damage scenarios are listed in Table 7, Table 8 and Table 9, respectively. Table 7, Table 8 and Table 9 also list the *MSE*, *R*^2^, and U95 of the SVM method to compare these two methods’ performance. From Table 5 and Table 6, with the increase in noise level, the two methods’ results all fluctuate around the true value. The errors are less than 10% except for two cases corresponding to Cable 1# in Scenario ④ and Cable 9# in Scenario ⑦, respectively, as shown in Table 6. For Scenario ⑦, the errors are large. The identified errors of the misidentified cables in Table 5 are all under 10% except that of Cable 10# of Scenario ⑦ that is 13.05% when the noise level is 15%. It indicates that even if there are misidentified cables, the identified results will be small.

As listed in Table 7, Table 8 and Table 9, *MSE* and U95 of the BVM method are close to 0, and *R*^2^ is close to 1. They all increase with the noise level. The *MSE*, *R*^2^, and U95 for Cables 3# and 9# of Scenario ⑦ are much greater than that of other scenarios. For Cable 3# of Scenario ⑦ with 15% measurement noise, the *R*^2^ value is 1.8755 which is greater than 1. That is because the damage severity of Cable 3# in Scenario ⑦ is 10% and the measurement noise is 15%. Furthermore, the maximum values of the *MSE* and U95 are 0.0080 and 0.0175 for this case, which shows the good performance of the method. Although damage severity of 25% has not been used in obtaining the function *de_i_*(*x*), the identification errors for this scenario are small and the corresponding *MSE* and U95 are close to 0, and *R*^2^ is close to 1. Meanwhile, for the SVM method [18], *MSE* and U95 are larger than 0, the smallest *MSE* and U95 are 0.0655 and 0.0502, and the largest *MSE* and U95 are 10.2385 and 0.6272, respectively. *R*^2^ is a little better than those of the BVM method, and is close to 1. In summary, this damage severity identification method has good performance, robustness, and strong anti-noise capability, and is better than the SVM method.

## 4. Conclusions

The bending strain-based BVM method has been developed to identify damaged cables in a cable-stayed bridge. The relationship between bending strain and the cable stiffness is derived by a force method. The FEM for the cable-stayed bridge is established using ANSYS and validated using field measurements. Furthermore, ***DIXV***, ***BVM***, and a test dataset are obtained using the validated FEM. Some conclusions are made as follows:

1. For a single-cable case, the damage severity does not have an effect on the ***BV***. Therefore, the ***BVM*** does not change with the cable damage severity, which is the key to the proposed BVM method.
2. The BVM method can directly identify single damaged cables and multiple damaged cables. With 100 samples, the sample probability of damaged cables is greater than 90%. The damage identification functions have a good performance to identify the cable damage severity. Therefore, the BVM method has good generalization and anti-noise capability.
3. The BVM method may be easily adapted to the field cable-stayed bridge health monitoring system. The identification probability could be improved with the increase in monitoring data.

Furthermore, further investigations on the sensitivity of this method to temperature variation, nonlinear vibration of the cable, vehicle–bridge coupling vibration, and different kinds of actual vehicle load are needed. More experimental data are also needed to further validate this method.

## Figures and Tables

**Table 1:** Comparison of the first five frequencies by the proposed model and experimental results.

Mode No.	Frequency (FEM) (Hz)	Frequency (Test) (Hz) [31]	Difference (%)	MAC
1	2.038	2.014	1.192	0.978
2	3.163	3.510	9.886	
3	4.088	3.645	12.154	
4	5.329	5.538	3.774	
5	6.530	6.068	7.614	

The difference = absolute (Frequency (FEM)—Frequency (Test))/Frequency (Test) × 100%.

**Table 2:** The hypothetical damage scenarios.

Damage Scenario	Damaged Cable Label	Damage Severity (%)	Remarks
①	1#	20	Single cable damaged
②	4#	10	
③	10#	25	
④	1#	20	Two cables damaged simultaneously
	4#	10	
⑤	2#	20	
	12#	20	
⑥	1#	10	Three cables damaged simultaneously
	4#	30	
	10#	20	
⑦	1#	30	Four cables damaged simultaneously
	3#	10	
	9#	20	
	11#	30	

**Table 3:** Results of identified damaged cables by a set of DIXVs for each damage scenario using the BVM method and the SVM method [18].

Noise Level	Method	Scenario ①	Scenario ②	Scenario ③	Scenario ④	Scenario ⑤	Scenario ⑥	Scenario ⑦
0%	BVM	1	4	10	1, 4	2, 12	1, 4, 10	1, 2, 3, 9, 10, 11
	SVM	1	4	10	1, 4	2, 12	——	——
5%	BVM	1	4	10	1, 4	2, 12	1, 4, 10	1, 2, 3, 9, 10, 11
	SVM	1	4	10	1, 4	2, 12	——	——
10%	BVM	1	4	10	1, 4	2, 12	1, 4, 10	1, 2, 3, 9, 11
	SVM	1	4	10	1, 4	2, 12	——	——
15%	BVM	1	4	10	1, 4	1, 2, 3, 12	1, 4, 10	1, 3, 9, 10, 11
	SVM	1	4	10	1, 4	2, 12	——	——
20%	BVM	1	4	10	1, 4	2, 11, 12	1, 3, 4, 9, 10	1, 3, 9, 10, 11
	SVM	1	4	10	1, 4	2, 12	——	——

“——” means this item does not exist. The values in the blue zone are the identified damaged cables using the SVM method [18].

**Table 4:** The damage severity identification function, when a single cable is damaged.

Damaged Cable Label	Damage Severity Identification Function
1#(9#)	de_1_(x) =1.268 × 10^−07^x^3^ − 3.624×10^−05^x^2^ + 0.006903x − 4.684×10^−17^
2#(10#)	de_2_(x) = 9.334 × 10^−08^x^3^ − 2.985×10^−05^x^2^ + 0.006615x + 4.372×10^−17^
3#(11#)	de_3_(x) = 2.758 × 10^−08^x^3^ − 1.341×10^−05^x^2^ + 0.004704x + −6.592×10^−17^
4#(12#)	de_4_(x) = 1.148 × 10^−08^x^3^ − 7.828×10^−06^x^2^ + 0.004198x + −3.504×10^−17^

**Table 5:** The damage severity identification results using the BVM method and the SVM method [18].

The Damage Scenario	Damaged Cable	Exact Damage Severity (%)	Identified Damage Severity (%)					
			Method	Noise 0%	Noise 5%	Noise 10%	Noise 15%	Noise 20%
①	1#	20	BVM	19.99	20.26	18.14	22.15	22.80
			SVM	19.43	19.17	20.25	23.57	19.28
②	4#	10	BVM	10.00	9.76	10.81	7.64	5.78
			SVM	10.14	10.29	9.36	9.94	9.34
③	9#	25	BVM	25.00	26.37	24.46	29.71	29.02
			SVM	24.73	25.01	23.04	23.06	26.09
④	1#	20	BVM	19.99	22.09	20.88	13.43	30.13
			SVM	19.28	19.32	20.07	19.61	23.80
	4#	10	BVM	10.01	10.26	9.78	11.69	12.32
			SVM	7.92	8.16	8.68	7.81	5.06
⑤	2#	20	BVM	20.41	20.91	20.67	21.13	16.17
			SVM	19.16	19.34	19.48	18.39	13.05
	12#	20	BVM	19.70	19.46	22.86	19.83	17.47
			SVM	19.31	18.21	20.21	19.39	19.73
	***1#***	——	BVM	——	——	——	***5.36***	——
	***3#***	——	BVM	——	——	——	***3.42***	——
	***11#***	——	BVM	——	——	——	——	***3.00***
⑥	1#	10	BVM	9.41	9.64	10.46	9.22	8.11
	4#	30	BVM	29.73	28.64	28.95	26.48	34.61
	10#	20	BVM	20.66	20.87	22.81	27.99	15.87
	***3#***	——	BVM	——	——	——	——	***9.60***
	***9#***	——	BVM	——	——	——	——	***3.91***
⑦	1#	30	BVM	28.02	26.44	21.01	25.35	30.31
	3#	10	BVM	6.78	6.42	3.82	11.75	9.93
	9#	20	BVM	15.00	15.31	8.59	13.11	22.74
	11#	30	BVM	28.26	28.73	27.63	22.30	30.58
	***2#***	——	BVM	***4.08***	***7.48***	***5.50***	——	——
	***10#***	——	BVM	***4.69***	***3.52***	——	***13.05***	***5.78***

The bold italics indicate the misidentified damaged cable labels and their identified damage severity. “——” means this item does not exist. The values in the pink zone are the identified damage severity using the SVM method [18].

**Table 6:** The error of the identified damage severity using the BVM method and the SVM method [18].

The Damage Scenario	Damaged Cable	Exact Damage Severity (%)	Error of Identified Damage Severity (%)					
			Method	Noise 0%	Noise 5%	Noise 10%	Noise 15%	Noise 20%
①	1#	20	BVM	−0.01	0.26	−1.86	2.15	2.8
			SVM	−0.57	−0.83	0.25	3.57	−0.72
②	4#	10	BVM	0	−0.24	0.81	−2.36	−4.22
			SVM	0.14	0.29	−0.64	−0.06	−0.66
③	10#	25	BVM	0	1.37	−0.54	4.71	4.02
			SVM	−0.27	0.01	−1.96	−1.94	1.09
④	1#	20	BVM	−0.01	2.09	0.88	−6.57	10.13
			SVM	−0.72	−0.68	0.07	−0.39	3.80
	4#	10	BVM	0.01	0.26	−0.22	1.69	2.32
			SVM	−2.08	−1.84	−1.32	−2.19	−4.94
⑤	2#	20	BVM	0.41	0.91	0.67	1.13	−3.83
			SVM	−0.84	−0.66	−0.52	−1.61	−6.95
	12#	20	BVM	−0.3	−0.54	2.86	−0.17	−2.53
			SVM	−0.69	−1.79	0.21	−0.61	−0.27
⑥	1#	10	BVM	−0.59	−0.36	0.46	−0.78	−1.89
	4#	30	BVM	−0.27	−1.36	−1.05	−3.52	4.61
	10#	20	BVM	0.66	0.87	2.81	7.99	−4.13
⑦	1#	30	BVM	−1.98	−3.56	−8.99	−4.65	0.31
	3#	10	BVM	−3.22	−3.58	−6.18	1.75	−0.07
	9#	20	BVM	−5	−4.69	−11.4	−6.89	2.74
	11#	30	BVM	−1.74	−1.27	−2.37	−7.7	0.58

The values in the pink zone are the errors of the identified damage severity using the SVM method [18].

**Table 7:** The MSE of the identified damaged severity of the 7 damage scenarios’ test dataset using the BVM method and the SVM method [18].

The Damage Scenario	Damaged Cable	Exact Damage Severity (%)	MSE of Identified Damage Severity				
			Method	Noise 5%	Noise 10%	Noise 15%	Noise 20%
①	1#	20	BVM	0.0001	0.0006	0.0013	0.0019
			SVM	0.7590	1.891	3.889	5.9577
②	4#	10	BVM	0.0000	0.0001	0.0003	0.0005
			SVM	0.0655	0.2088	0.3985	1.1179
③	10#	25	BVM	0.0003	0.0013	0.0024	0.0041
			SVM	0.6353	2.4003	4.7062	10.0898
④	1#	20	BVM	0.0001	0.0005	0.0013	0.0018
			SVM	1.0166	2.4357	4.3462	5.9171
	4#	10	BVM	0.0000	0.0001	0.0003	0.0005
			SVM	4.1723	4.5239	5.0131	5.5097
⑤	2#	20	BVM	0.0002	0.0007	0.0022	0.0040
			SVM	1.0083	1.5974	2.9434	4.7203
	12#	20	BVM	0.0001	0.0005	0.0011	0.0016
			SVM	1.0372	3.5539	6.3826	10.2385
⑥	1#	10	BVM	0.0001	0.0003	0.0006	0.0014
	4#	30	BVM	0.0002	0.0011	0.0015	0.0036
	10#	20	BVM	0.0003	0.0008	0.0019	0.0039
⑦	1#	30	BVM	0.0007	0.0013	0.0044	0.0046
	3#	10	BVM	0.0011	0.0014	0.0023	0.0021
	9#	20	BVM	0.0033	0.0037	0.0080	0.0076
	11#	30	BVM	0.0009	0.0017	0.0035	0.0055

The values in the pink zone are *MSE* of the identified damage severity using the SVM method [18].

**Table 8:** The *R*^2^ of the identified damaged severity of the 7 damage scenarios’ test dataset using the BVM method and the SVM method [18].

The Damage Scenario	Damaged Cable	Exact Damage Severity (%)	*R*^2^ of Identified Damage Severity				
			Method	Noise 5%	Noise 10%	Noise 15%	Noise 20%
①	1#	20	BVM	1.0028	1.0154	1.0319	1.0484
			SVM	1.0020	1.0050	1.0101	1.1056
②	4#	10	BVM	1.0028	1.0096	1.0296	1.0516
			SVM	1.0006	1.0020	1.0039	1.0107
③	10#	25	BVM	1.0053	1.0203	1.0390	1.0720
			SVM	1.0010	1.0039	1.0075	1.0166
④	1#	20	BVM	1.0032	1.0114	1.0334	1.0470
			SVM	1.0028	1.0065	1.0116	1.0154
	4#	10	BVM	1.0033	1.0091	1.0255	1.0524
			SVM	1.0701	1.0768	1.0864	1.0927
⑤	2#	20	BVM	1.0055	1.0181	1.0541	1.1033
			SVM	1.0028	1.0043	1.0080	1.0129
	12#	20	BVM	1.0038	1.0128	1.0305	1.0412
			SVM	1.0028	1.0095	1.0180	1.0269
⑥	1#	10	BVM	1.0156	1.0297	1.0738	1.1627
	4#	30	BVM	1.0028	1.0122	1.0177	1.0420
	10#	20	BVM	1.0062	1.0192	1.0488	1.0982
⑦	1#	30	BVM	1.0085	1.0167	1.0622	1.0556
	3#	10	BVM	1.2923	1.3935	1.8755	1.6798
	9#	20	BVM	1.1725	1.1846	1.4798	1.3949
	11#	30	BVM	1.0113	1.0213	1.0467	1.0714

The values in the pink zone are *R*^2^ of the identified damage severity using the SVM method [18].

**Table 9:** The U95 of the identified damaged severity of the 7 damage scenarios’ test dataset using the BVM method and the SVM method [18].

The Damage Scenario	Damaged Cable	Exact Damage Severity (%)	U95 of Identified Damage Severity				
			Method	Noise 5%	Noise 10%	Noise 15%	Noise 20%
①	1#	20	BVM	0.0021	0.0048	0.0071	0.0087
			SVM	0.1708	0.2696	0.3865	0.4784
②	4#	10	BVM	0.0010	0.0019	0.0033	0.0045
			SVM	0.0502	0.0896	0.1237	0.2072
③	10#	25	BVM	0.0036	0.0072	0.0096	0.0126
			SVM	0.1562	0.3037	0.4252	0.6226
④	1#	20	BVM	0.0022	0.0042	0.0071	0.0083
			SVM	0.1976	0.3059	0.4086	0.4768
	4#	10	BVM	0.0011	0.0019	0.0032	0.0045
			SVM	0.4004	0.4169	0.4388	0.4601
⑤	2#	20	BVM	0.0030	0.0053	0.0091	0.0124
			SVM	0.1968	0.2477	0.3363	0.4258
	12#	20	BVM	0.0024	0.0044	0.0066	0.0079
			SVM	0.1996	0.3695	0.4952	0.6272
⑥	1#	10	BVM	0.0023	0.0033	0.0050	0.0073
	4#	30	BVM	0.0031	0.0064	0.0077	0.0117
	10#	20	BVM	0.0031	0.0056	0.0086	0.0122
⑦	1#	30	BVM	0.0051	0.0072	0.0130	0.0133
	3#	10	BVM	0.0064	0.0073	0.0094	0.0090
	9#	20	BVM	0.0112	0.0119	0.0175	0.0171
	11#	30	BVM	0.0058	0.0081	0.0115	0.0145

The values in the pink zone are U95 of the identified damage severity using the SVM method [18].

## Data Availability

Data is unavailable due to privacy restrictions.
